# Ruxolitinib Pharmacokinetics and Exposure–Toxicity Relationship in Hematologic Malignancies and Immune‐Mediated Diseases: A Prospective Observational Study

**DOI:** 10.1002/cpt.70367

**Published:** 2026-06-24

**Authors:** Jérémie Tachet, Sara C. Meyer, Francesco Grandoni, Emmanuel Haefliger, Monika Nagy‐Hulliger, Jörg P. Halter, Jakob R. Passweg, David Haefliger, Carine Bardinet, Laurent A. Decosterd, Monia Guidi, François R. Girardin

**Affiliations:** ^1^ Service of Clinical Pharmacology, Department of Medicine Lausanne University Hospital and University of Lausanne Lausanne Switzerland; ^2^ Department of Hematology and Central Hematology Laboratory Inselspital, Bern University Hospital and University of Bern Bern Switzerland; ^3^ Service and Central Laboratory of Hematology Lausanne University Hospital and University of Lausanne Lausanne Switzerland; ^4^ Service of Hematology Hospital of Morges Morges Switzerland; ^5^ Division of Hematology Basel University Hospital and University of Basel Basel Switzerland; ^6^ Laboratory of Clinical Pharmacology, Department of Laboratory Medicine and Pathology Lausanne University Hospital and University of Lausanne Lausanne Switzerland; ^7^ Center for Research and Innovation in Clinical Pharmaceutical Sciences Lausanne University Hospital and University of Lausanne Lausanne Switzerland; ^8^ Institute of Pharmaceutical Sciences of Western Switzerland University of Geneva, University of Lausanne Geneva, Lausanne Switzerland

## Abstract

Ruxolitinib pharmacokinetics (PK) has been characterized in clinical trials but remains poorly documented in real‐world practice. This project aimed to investigate ruxolitinib PK in routine clinical practice, identify factors driving its variability, and explore exposure–response relationships to assess the potential role of therapeutic drug monitoring. In total, 221 steady‐state ruxolitinib concentrations from 77 adult patients enrolled across several centers in Switzerland were analyzed. Demographic and clinical data were recorded at each visit. Population pharmacokinetic (popPK) analysis was performed with MonolixSuite® 2024R1 (Lixoft, France). Model‐based simulations were used to predict trough concentration (*C*_min_) and the area under the concentration–time curve over 24 h (AUC_24_) for the recommended dosage regimens, as a function of covariates. An exploratory pharmacokinetic/pharmacodynamic analysis was conducted in the overall cohort. Ruxolitinib PK was characterized by a marked between‐subject variability in clearance (45%). Strong cytochrome (CYP) 3A4 or dual CYP2C9/CYP3A4 inhibitors reduced clearance by 39%. At 10 mg twice daily, model‐based simulations showed CYP inhibitors increased median *C*_min_ and AUC_24_ by 2.9‐ and 1.7‐fold, respectively. Still, no significant exposure–efficacy relationship was identified. However, higher ruxolitinib exposure tended to be associated with increased toxicity in the overall population (*C*_min_ and AUC_24_, *P*≈0.02–0.03). In clinical practice, ruxolitinib exposure shows substantial variability and is strongly affected by strong CYP inhibitors that are frequently co‐administered. Whilst no clear exposure–efficacy relationship was observed, higher exposure was linked to increased toxicity risk, arguing for careful dose adjustment.

**Study Highlights**

**WHAT IS THE CURRENT KNOWLEDGE ON THE TOPIC?**

Ruxolitinib pharmacokinetics (PK) has been characterized in clinical trials. However, PK and exposure–response relationships remain poorly documented in routine clinical practice.

**WHAT QUESTION DID THIS STUDY ADDRESS?**

This study aimed to characterize the pharmacokinetic profile of ruxolitinib in a heterogeneous real‐world cohort, identify key factors of variability, and explore exposure–response relationships to evaluate the potential clinical utility of therapeutic drug monitoring (TDM).

**WHAT DOES THIS STUDY ADD TO OUR KNOWLEDGE?**

Ruxolitinib clearance showed moderate‐to‐high between‐subject variability, and strong CYP inhibitors significantly reduced clearance. This resulted in substantially higher exposure, which was associated with increased toxicity risk. Simulations suggested that patients receiving strong CYP inhibitors may exceed a previously proposed trough concentration toxicity threshold.

**HOW MIGHT THIS CHANGE CLINICAL PHARMACOLOGY OR TRANSLATIONAL SCIENCE?**

These findings support closer attention to drug–drug interactions and individualized dose adjustment in routine care to improve ruxolitinib safety, particularly in patients receiving strong CYP inhibitors or with other factors that alter metabolism.

Myeloproliferative neoplasms (MPN) are clonal hematopoietic stem cell disorders characterized by abnormal proliferation of one or more myeloid lineages, which include Philadelphia chromosome (Ph)‐positive or Ph‐negative neoplasms. The most common Ph‐negative subtypes are polycythemia vera (PV), essential thrombocythemia (ET), and myelofibrosis (MF).^1^ Constitutive activation of the Janus kinase/signal transducer and activator of transcription (JAK–STAT) pathway, most commonly driven by the JAK2^V617F^ mutation, is a central feature of the three subtypes of MPN Ph‐negative pathophysiology, leading to altered hematopoiesis and cytokine signaling.^2^ Beyond MPN, the JAK–STAT pathway also plays a key role in graft‐versus‐host disease (GvHD), a major and potentially fatal complication of allogeneic hematopoietic stem cell transplantation.^3^, ^4^, ^5^, ^6^

Ruxolitinib is a JAK1/2 inhibitor used orally for the treatment of intermediate‐ or high‐risk MF (including primary MF, post–PV and post–ET MF) and PV in adults with inadequate response or intolerance to first‐line cytoreductive treatment, as well as for steroid‐refractory acute GvHD and chronic GvHD after failure of one or two lines of systemic therapy in patients ≥ 12 years of age.^7^ In addition to systemic use, a topical formulation is approved for the treatment of nonsegmental vitiligo and mild‐to‐moderate atopic dermatitis.^8^ Beyond its approved indications, ruxolitinib is also employed off‐label in selected immune‐mediated and hematologic conditions, such as the VEXAS (vacuoles, E1 enzyme, X‐linked, autoinflammatory, somatic) syndrome,^9^ autoimmune polyendocrinopathy–candidiasis–ectodermal dystrophy (APECED) syndrome,^10^ and hemophagocytic lymphohistiocytosis (HLH).^11^

Ruxolitinib is usually administered orally twice daily, with doses typically ranging from 5 to 25 mg according to indication and hematological values. Its safety profile is mainly characterized by hematologic toxicity, specifically anemia, neutropenia, and thrombocytopenia, and by an increased risk of infections.^12^, ^13^, ^14^ To date, the pharmacokinetics (PK) of ruxolitinib have been described primarily from drug development studies in healthy volunteers and phase I–III trial populations.^15^, ^16^, ^17^, ^18^, ^19^ Dose‐proportional PK have been reported over a wide dose range (5–200 mg), with time to peak concentrations (*T*_max_) reached within 1–2 hours and a short elimination half‐life (*t*_1/2_) of 3–6 hours.^15^ Ruxolitinib is extensively metabolized by cytochrome P450 (CYP) 3A4, with a minor contribution from CYP2C9. It generates several active metabolites (primarily M18 and M27) which account for approximately 25% and 11% of the parent drug area under the curve (AUC). These metabolites retain JAK1/JAK2 inhibitory activity, although with a two‐ to five‐fold lower potency compared with the parent compound.^20^ Collectively, they contribute approximately 18% to its overall pharmacological activity. Most of the drug is excreted in the urine, primarily as metabolites, with a smaller amount excreted in the feces. Although not associated with distinct toxicities, increased exposure to these metabolites, such as in patients with renal impairment, may enhance the overall pharmacodynamic activity.^18^ Furthermore, its exposure is susceptible to drug–drug interactions, particularly with azole antifungals frequently used in MPN and GvHD for antifungal prophylaxis.^7^, ^16^, ^21^, ^22^, ^23^ In addition, inflammation‐driven suppression of CYP activity known as phenoconversion and its reversal with cytokine‐lowering therapies may further modify ruxolitinib and co‐medication exposure.^24^, ^25^, ^26^, ^27^, ^28^, ^29^ Given its short *t*_½_, steady‐state concentrations are typically reached within 48 hours of repeated dosing. Hepatic impairment reduces clearance (CL) and prolongs *t*_1/2_, whereas renal impairment has little effect on the parent drug AUC but may increase the pharmacological effect through accumulation of active metabolites.^18^, ^20^

In phase II–III studies, population pharmacokinetic (popPK) analyses in MF patients identified sex and body weight (BW) as statistically significant covariates on CL and volume, respectively.^17^ However, the magnitude of these effects was considered too small to justify dose adjustment in adults, supporting the original dosing recommendations. In contrast, in GvHD patients treated in routine care, ruxolitinib CL was approximately 50% lower than in MF, resulting in markedly higher exposure. Co‐administration of strong CYP3A4 or CYP2C9 inhibitors further reduced CL by about 15%.^30^ Exposure–efficacy and exposure–toxicity relationships have been established during pivotal trials, with higher ruxolitinib exposure associated with improved spleen volume reduction and symptom control in MF, but also with an increased incidence of hematological toxicity.^20^

These findings from highly selected clinical trial populations may not reflect the heterogeneity of real‐world patients treated with ruxolitinib in everyday practice, including those with comorbidities, organ impairment, or co‐medications that can alter drug exposure. In this context, therapeutic drug monitoring (TDM) could help detect under‐ or overexposure and guide dose adjustment. Although a formal therapeutic range has not been established, a previous study in a small cohort of GvHD patients identified a trough concentration (*C*_min_) cut‐off of 21 ng/mL as a discriminator for increased risk of adverse events.^30^ These observations underscore the need to characterize ruxolitinib PK in larger real‐world cohorts to quantify between‐patient variability and evaluate the potential added value of TDM in complex patients.

This study is part of an ongoing observational project designed to prospectively monitor patients with MPN, GvHD, and other hematologic disorders receiving ruxolitinib in routine clinical practice. Our primary objective was to characterize the PK profile of ruxolitinib, quantify its variability, and assess the impact of demographic, clinical, and concomitant medications on drug exposure using a population approach. We also conducted exploratory analyses of exposure–response relationships to assess the potential role of TDM.

## MATERIALS AND METHODS

### Study population and design

This prospective observational study was conducted between August 2023 and November 2025 in Switzerland, following protocol approval by the Research Ethics Committee of the Canton of Vaud (2023‐00904).^31^ All procedures complied with the Declaration of Helsinki^32^ and applicable national regulations.^33^, ^34^ Samples were collected prospectively from patients receiving ruxolitinib in several Swiss hospitals (University Hospital of Bern, University Hospital of Basel, Hospital of Morges, and University Hospital of Lausanne) and private practices. Adult patients (≥ 18 years) initiating or already receiving ruxolitinib were eligible if capable of judgment and able to provide informed consent. Blood samples were obtained at steady‐state and at random times after the last drug intake during routine outpatient visits. A subgroup of patients took part in a detailed PK sub‐study, which included one pre‐dose sample and seven post‐dose samples after a ruxolitinib administration over a period of 8 hours. Clinical and demographic data were obtained from medical records and a dedicated case report form, which also captured the date and time of the last dose and blood sampling. Collected variables included age, sex, BW, height, dosage, underlying disease, co‐medications, C‐reactive protein (CRP), creatinine, and physician global assessment (PhGA) score, categorized into severe, moderate, low, and no disease activity. Adverse events reported by the patient during the visit were documented according to the Common Terminology Criteria for Adverse Events v5.0. Concomitant medications were screened for potential CYP‐mediated interactions with ruxolitinib according to drug interactions tool and literature.^7^, ^16^, ^35^ As no weak inhibitors or inducers were identified in the study population, they were classified as moderate CYP3A inhibitors or strong CYP inhibitors (including strong CYP3A inhibitors, dual CYP2C9/CYP3A inhibitors).

### Analytical method

Ruxolitinib plasma concentrations were quantified at the Lausanne University Hospital using a validated multiplex liquid chromatography coupled to tandem mass spectrometry method with lower limit of quantification at 0.5 ng/mL, using a previously published validated assay.^36^

### Population pharmacokinetic analysis

Analyses were performed using MonolixSuite® 2024R1 (Lixoft, France). Data management and statistical analysis was performed using R studio (version 2023.09.1, Integrated Development for R. RStudio, PBC, Boston, Massachusetts, USA, http://www.rstudio.com/). Samples with missing or unreliable timing information were excluded from the popPK analysis. Observations below the lower limit of quantification were handled as censored data in the likelihood function.

#### Base and covariate models

A classical stepwise model‐building approach was used to identify the popPK model that best described ruxolitinib concentrations. Several candidate models with first‐order absorption and linear elimination were evaluated, including one‐ and two‐compartment structures. The absorption rate constant (*k*_a_) was either fixed or estimated depending on model stability. When fixed, its value was calculated using published time to peak concentration (*T*_max_) values. A log‐normal distribution was assumed for all parameters, and between‐subjects variability was sequentially tested for all of them. Additive, proportional, and mixed effect error models were evaluated to select the best residual unexplained variability.

Biologically plausible covariates with < 10% missing values were first evaluated individually on the base model using a classical stepwise forward inclusion approach. Continuous covariates (BW, BMI, age, height) and categorical covariates (sex, interacting drugs, underlying disease) were tested for significance on ruxolitinib PK. Covariate effects were evaluated on all PK parameters, including those without between‐subject variability, by testing them with either a fixed or an estimated allometric exponent. Statistically significant relationships identified during forward inclusion were then combined, and only the most significant associations were retained before applying a backward elimination step to identify the final model.

The effects of the continuous covariates were evaluated using log–log models as follows:

$$\log\left(P_{i}\right)=\log\left(P_{\mathrm{pop}}\right)+\beta_{\mathrm{cov}}\cdot \log\left(\frac{{\mathrm{cov}}_{i}}{{\mathrm{cov}}_{\text{median}}}\right)+\eta_{i}$$

where ${\mathrm{cov}}_{i}$ and ${\mathrm{cov}}_{\text{median}}$ are the individual and population median covariate values, $P_{i}$ is the individual predicted PK parameter for the $i^{\mathrm{th}}$ subject, $P_{\mathrm{pop}}$ is the typical population PK parameter, $\beta_{\mathrm{cov}}$ represents the fixed effect of the covariate, $\eta_{i}$ accounts for the between‐subject variability term, assumed to be normally distributed with mean zero and variance $\omega^{2}$.

Categorical covariates were included as binary or multi‐category variables. Dummy variables were used to code for the underlying diseases, grouped into the following categories: MPN (MF, PV, ET), GvHD, other hematological diseases (VEXAS syndrome, APECED syndrome, HLH), and missing data. CYP inhibitors were first evaluated as separate categories (no inhibitor vs. moderate CYP3A inhibitors vs. strong CYP3A and dual CYP3A/2C9 inhibitors vs. missing data) to assess their impact on ruxolitinib PK. Categories showing similar effects were subsequently pooled together (**Table** **S1**). Time‐varying covariates were incorporated as regressors, allowing covariate values to vary between sampling occasions within the same individual. Sensitivity analyses were performed to investigate the potential leverage effect of covariates with missing values and unreliable concentration measurements. This involved excluding such data and performing the popPK analysis on the resulting reduced dataset.

#### Model selection and evaluation

The difference between two nested models objective function values (−2·log‐likelihood, Δ2LL) was used to discriminate between them and to identify statistically significant improvements during the overall model building process. A drop of Δ2LL lower than −3.84 for one additional parameter was considered statistically significant (*P* ≤ 0.05) for model development and forward covariate testing. During backward deletion, a more stringent threshold was used, and covariates were retained only if their removal increased the Δ2LL by more than 6.63 (*P* < 0.01) for one degree of freedom. Non‐nested model comparisons were performed using the corrected Bayesian information criterion (∆BICc ≤ −2).

Model selection was further guided by the precision of parameter estimates, assessed by the relative standard error (RSE), the goodness‐of‐fit plots (observed vs. individual predictions; weighted residuals vs. time and predictions), and prediction‐corrected visual predictive checks (pcVPC, *n* = 1,000).^37^, ^38^, ^39^

The nonparametric bootstrap method (*n* = 2,000), as implemented in Monolix®, was used as internal validation to assess the reliability of the final model comparing original parameter estimates with the corresponding bootstrap medians and their 95% confidence intervals (CI_95%_).

### Model‐based simulations

We performed 1,000 simulations at steady state based on the final model incorporating between‐subject variability to predict *C*_min_, peak plasma concentration (*C*_max_), and the area under the concentration–time curve over 24 h (AUC_24_) for the recommended dosing regimens (5–25 mg twice daily) in a group of virtual subjects stratified according to identified covariate characteristics. Simulations accounted for the presence or absence of strong CYP3A or dual CYP2C9/CYP3A inhibitors. Individual BW values were randomly generated from a uniform distribution across our study population range.

### Exploratory pharmacokinetic/pharmacodynamic analysis

Exploratory pharmacokinetic/pharmacodynamic (PK/PD) analyses were conducted by predicting individual steady‐state *C*_min_, *C*_max_, and AUC_24_ at the time of each documented efficacy or toxicity assessment, using individual *post hoc* PK parameter estimates from the final population PK model. Efficacy was assessed using the PhGA score, dichotomized as severe vs. non‐severe disease activity, and toxicity was defined as the occurrence of any adverse event. Duplicate records with identical exposure metrics (*C*_min_, *C*_max_, and AUC_24_) and unchanged efficacy or toxicity status were removed from the analysis. Patients with overlapping diagnoses were assigned to a single category based on clinical judgment. Exposure–response relationships were explored using logistic regression models with log‐transformed PK metrics as predictors. Effects were expressed as odds ratios with 95% confidence intervals. All analyses were conducted in R.

## RESULTS

### Study population

**Figure 1:**
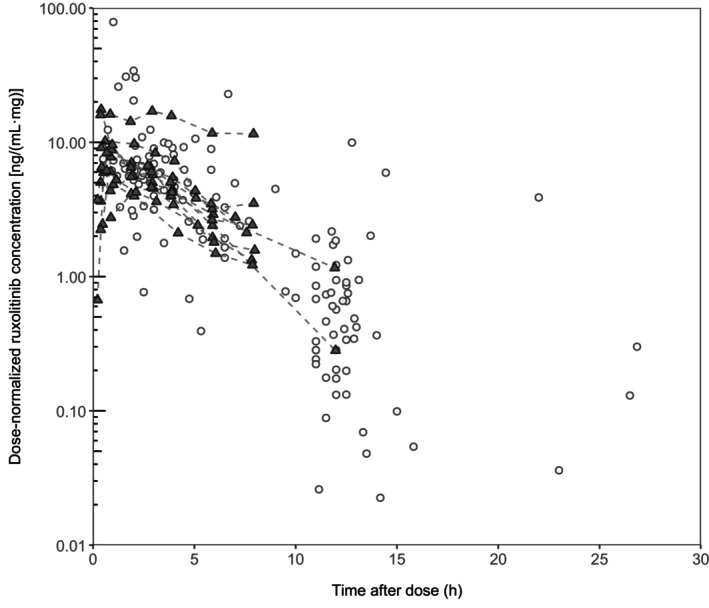
Observed ruxolitinib plasma concentrations normalized to individual daily dose. Concentrations are shown on a logarithmic scale. Sparse samples are displayed as open circles, whereas concentrations from the detailed PK sub‐study are shown as gray triangles connected by a dashed black line. Plots display observations up to 30 h after dosing.

Overall, 221 measurements of ruxolitinib plasma concentrations, including 12 below the limit of quantification, collected from 77 patients were available for the analysis. Of these, 11 patients enrolled in the intensive PK sub‐study contributed 87 plasma concentrations (**Figure** **1**). Most patients provided 1 (*n* = 36, 47%) or 2 (*n* = 19, 25%) samples, whereas the remaining (*n* = 22, 28%) contributed between 3 and 11 samples. Ruxolitinib dosing regimens were highly heterogeneous, ranging from 5 to 25 mg once and twice daily to alternative schedules, such as alternating doses within a day or administration every 48 or 72 hours (**Table** **S2**). **Table** **1** presents the characteristics of the study population. Details on adverse events are reported in **Table** **S3**.

**Table 1:** Demographic and clinical characteristics of the study population

Summary of patient characteristics	PK analysis	
	Median [range] or *n* (%)	Missing data *n* (%)
Demographic characteristics		
Age (years)	64 [17–89]	0 (0)
Male	46 (60)	0 (0)
Female	31 (40)	0 (0)
Body weight (kg)	72.0 [40.7–116.0]	0 (0)
Height (cm)	172 [146–202]	0 (0)
BMI (kg/m^2^)	24.7 [15.4–33.2]	0 (0)
Underlying diseases		
GvHD group		2 (1)
GvHD	33 (43)	
MPNs subtype		
MF	23 (30)	
PMF	10 (13)	
Post‐ET MF	4 (5)	
Post‐PV MF	8 (10)	
MDS/MPN‐RS‐T	1 (1)	
PV	12 (16)	
ET	2 (3)	
Other hematologic disease group		
VEXAS syndrome	3 (4)	
APECED syndrome	1 (1)	
HLH	1 (1)	
Reported interacting drug^a^		
Moderate CYP3A inhibitors^b^	8 (4)	8 (4)
Strong CYP3A inhibitor^c^, Dual CYP2C9 and CYP3A inhibitor^d^	24 (11)	
Adverse events^e^		
Adverse events	101 (48)	12 (5)
Clinical response^f^		
No activity	12 (5)	32 (14)
Weak activity	118 (53)	
Moderate activity	33 (15)	
High activity	26 (12)	

APECED, Autoimmune polyendocrinopathy‐candidiasis‐ectodermal dystrophy; BMI, body mass index; ET, essential thrombocythemia; GvHD, Graft‐versus‐host disease (acute and chronic); HLH, hemophagocytic lymphohistiocytosis; MDS/MPN‐RS‐T, myelodysplastic/myeloproliferative neoplasms with ring sideroblasts and thrombocytosis; MF, myelofibrosis; MPN, myeloproliferative neoplasms; PMF, primary myelofibrosis; Post‐ET MF, post‐essential thrombocythemia myelofibrosis; Post‐PV MF, post‐polycythemia vera myelofibrosis; PV, polycythemia vera; VEXAS, Vacuoles, E1 Enzyme, X‐linked, Autoinflammatory, and Somatic Syndrome.

^a^ Number of samples for which at least one interacting drug was documented at the time of sampling.

^b^ Includes isavuconazole, letermovir, and verapamil.

^c^ Includes posaconazole.

^d^ Includes fluconazole.

^e^ Number of samples for which at least one adverse event was documented at the time of sampling.

^f^ Number of samples for which Physician global assessment score was documented at the time of sampling.

### Structural, statistical, and covariate models

A one‐compartment model with first order absorption and elimination, including between‐subject variability associated with CL adequately described the ruxolitinib PK profile. Adding between‐subject variability on the volume of distribution (*V*) or using a two‐compartment model did not improve the fit (Δ2LL = −1.74 and −0.93, respectively; *P* > 0.05). The estimated parameters for the base model were *k*_*a*_ fixed to 3.24 h^−1^, a CL of 13.3 L/h (between‐subject variability, CV: 45%), and a *V* of 70.3 L, resulting in an estimated *T*_max_ of 0.92 h and *t*_1/2_ of 3.45 h, comparable to reported literature values.

**Table 2:** Final population pharmacokinetic parameter estimates of ruxolitinib with their bootstrap evaluations

Parameters	Final model		Bootstrap (*n* = 2,000)	
	Estimate	RSE (%)	Median	CI_95%_
*k*_*a*_ (h^−1^)	3.24 FIX		3.24 FIX	
*V*_pop_ (L)	69	5	71	[63–79]
*β*_V,BW_	1 FIX		1 FIX	
CL_pop_ (L)	14	6	14	[12–16]
*ω*_CL_ (CV, %)	37	13	34	[23–47]
*β*_CL,CYP3A/2C9 inhibitors_	−0.50	17	−0.48	[−0.87 to −0.04]
*σ*_prop_ (CV, %)	42	6	43	[35–52]

Final model: $\log\left(V_{i}\right)=\log\left(V_{\mathrm{pop}}\right)+\beta_{V,\mathrm{BW}}\cdot \log\left(\frac{{\mathrm{BW}}_{i}}{{\mathrm{BW}}_{\text{median}}}\right);\log\left({\mathrm{CL}}_{i}\right)=\log\left({\mathrm{CL}}_{\mathrm{pop}}\right)+\beta_{\mathrm{CL},\mathrm{CYP}3A/2C9\;\text{inhibitors}}\cdot 1_{\left\{\mathrm{CYP}3A/2C9\;\text{inhibitors}=1\right\}}+\eta_{\mathrm{CL},i}$.

BW_i_, individual body weight; BW_median_, the population median value of 73 kg; CI_95%_, 95% confidence interval; CL_i_, individual value of clearance in the *i*th subject; CL_pop_, typical clearance in the population; *k*_*a*_, typical first‐order absorption rate constant; RSE, relative standard error expressed as a percentage; *V*_i_, individual value of the volume of distribution in the *i*th subject; *V*_pop_, typical *V* in the population; $1_{\left\{\mathrm{CYP}3A/2C9\;\text{inhibitors}=1\right\}}$, indicator function that equals 1 when at leasst one strong CYP3A or dual CYP3A/2C9 inhibitor is present and 0 otherwise; $\beta_{\mathrm{CL},\mathrm{CYP}3A/2C9\;\text{inhibitors}}$, parameter for the strong CYP3A or dual CYP3A/2C9 inhibitor effect on CL; $\beta_{V,\mathrm{BW}}$, parameter for the body weight effect on *V*; $\eta_{{\mathrm{CL}}_{i}}$, individual between‐subject variability term, assumed to be normally distributed with mean zero and variance $\omega_{\mathrm{CL}}^{2}$ for CL; $\sigma_{\text{prop}}$, proportional residual error; $\omega_{\mathrm{CL}}$, between‐subjects variability on CL.

In univariate analyses, sex (Δ2LL = −5.04, *P* < 0.05), height (Δ2LL = −6.63, *P* < 0.01), BMI (Δ2LL = −10.8, *P* < 0.001) and BW (Δ2LL = −14.2, *P* < 0.01) were significantly associated with *V*. The exponent of the BW and *V* relationships was fixed to 1, as allowing the exponent to vary did not significantly improve model fit (ΔBIC = −13.6). Strong CYP3A and dual CYP2C9/CYP3A inhibitors were associated with a significant reduction in ruxolitinib CL (Δ2LL = −12.1, *P* < 0.001). No other covariates had a statistically significant effect on CL. **Table** **S1** and **Figure** **S1** summarize the univariate analyses, the forward inclusion and backward elimination steps of the covariate analyses, respectively. In the final model (**Table** **2**), BW was retained on *V* (exponent fixed to 1) and strong CYP inhibitors and dual CYP2C9/CYP3A were retained on clearance. Patients receiving at least one strong CYP3A or one dual CYP2C9/CYP3A inhibitor had a 39% reduction in CL. Covariates included in the final popPK model explained approximately 30% of the between‐subject variability on CL.

### Model evaluation

The final model was successfully validated. The goodness‐of‐fit diagnostic plots (**Figure** **S2**) and the pcVPC (**Figure** **S3**) support its predictive performance. Approximately 96% of the prediction‐corrected data were within the pcVPC limits. Shrinkage for CL was low (1.6%) in the final model. Bootstrap results demonstrated good precision of the final model parameters, with all point estimates lying within the 95% confidence intervals of their bootstrap median estimates (**Table** **2**) and a maximum relative bias of 6%.

### Model‐based simulations

**Figure 2:**
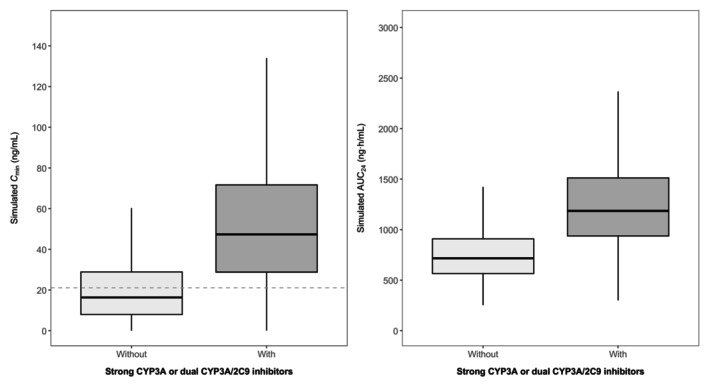
Simulated steady‐state *C*_min_ and AUC_24_ of ruxolitinib for 10 mg twice daily, with and without strong CYP3A or dual CYP3A/2C9 inhibitor. Boxes show the median and interquartile range from the 25th percentile to the 75th percentile; whiskers extend to 1.5 times the interquartile range. The dashed gray line indicates the *C*_min_ previously identified toxicity threshold of 21 ng/mL.^30^ AUC_24_, area under the curve over the dosing interval of 24 h; *C*_min_, trough concentration; CYP, cytochrome P450.

**Figure** **2** illustrates the steady‐state predicted *C*_min_, *C*_max_, and AUC_24_ in virtual individuals receiving 10 mg twice daily, with and without strong CYP3A and dual CYP2C9/CYP3A inhibitors. When co‐administered, median steady‐state *C*_min_ of ruxolitinib increased by 2.9‐fold, AUC_24_ by 1.7‐fold, and *C*_max_ by 1.3‐fold compared with ruxolitinib administration alone.

**Table** **3** presents the proportion of patients exceeding the *C*_min_ cut‐off of 21 ng/mL, a level previously reported as predictive of a higher risk of adverse events.^30^ Interestingly, the simulation results for the standard 10 mg twice daily dosing regimen show that 39% of virtual subjects without and 85% with strong CYP3A or dual CYP3A/2C9 inhibitor exceed this threshold.

**Table 3:** Percentage of patients with trough concentrations above the reported *C*_min_ toxicity threshold

Dosing regimen	Strong CYP3A or dual CYP3A/2C9 inhibitor	Median *C*_min_ (ng/mL)	PI_90%_	Patients with *C*_min_ > 21 ng/mL^a^ (%)
5 mg BID	−	7.5	[0.8–28.0]	11
	+	23	[6–61]	55
10 mg BID	−	16	[2–55]	39
	+	48	[11–119]	85
15 mg BID	−	25	[2–87]	56
	+	69	[18–174]	94
20 mg BID	−	32	[3–118]	65
	+	94	[22–235]	95
25 mg BID	−	39	[4–130]	74
	+	115	[24–298]	96

BID, twice daily; *C*_min_, Trough concentration; CYP, cytochrome P450; PI_90%_, 90% prediction interval.

^a^ The toxicity threshold was identified in GvHD patients as discriminant for a higher risk of adverse events.^30^

### Exploratory PK/PD analysis

**Figure 3:**
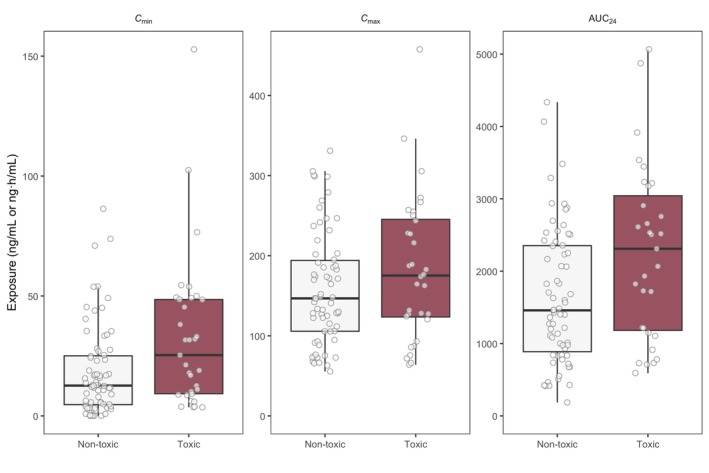
Exposure–toxicity relationship for ruxolitinib in the overall population. Boxplots show model‐predicted steady‐state *C*_min_, *C*_max_, and AUC_24_ according to the absence or presence of any adverse event. Points represent individual visit‐level observations; boxes indicate median and interquartile range, and whiskers extend to 1.5× IQR. AUC_24_, area under the curve over the dosing interval of 24 h; *C*_max_, peak plasma concentrations; *C*_min_, trough concentrations.

Exploratory PK/PD analysis showed no significant association between exposure and severe disease activity in the overall population (*n* = 75, *P* ≥ 0.49) (**Figure** **S4**). In contrast, higher ruxolitinib exposure was associated with an increased risk of toxicity. A two‐fold increase in *C*_min_ and AUC_24_ was associated with 1.5‐fold (OR: 1.51, 95% CI: 1.07–2.13, *P* = 0.019) and 1.9‐fold (OR: 1.88, 95% CI: 1.08–3.29, *P* = 0.026) higher odds of toxicity, respectively, whereas *C*_max_ was not significantly associated with this endpoint (*P* = 0.25) (**Figure** **3**). The data did not allow identification of a robust exposure threshold to discriminate patients who experienced toxicity from those who did not.

## DISCUSSION

In this prospective real‐world study, we characterized the PK of ruxolitinib and explored exposure–response relationships in a heterogeneous cohort of patients with MPN, GvHD, and other hematologic disorders treated with a wide range of dosing regimens, using both sparse and rich sampling. Only a few popPK studies of ruxolitinib have been published to date,^17^ and real‐world evidence remains particularly limited.^30^, ^40^ In phase II and III studies, a two‐compartment model with first‐order absorption and a lag time adequately described the full ruxolitinib PK profile.^17^ In contrast, we found that a one‐compartment model with first‐order absorption and linear elimination, including between‐subject variability on CL, adequately described the observed concentrations, consistent with findings from another real‐world study.^40^ The final model yielded *T*_max_ and *t*_1/2_ estimates in line with previously published studies,^17^, ^30^ despite differences in the structural model. We observed moderate‐to‐high between‐subject variability in CL, indicating considerable heterogeneity in drug exposure across patients.

Between‐subject variability was partly explained by the effect of BW on the *V* and by the impact of strong CYP3A and dual CYP3A/2C9 inhibitors on ruxolitinib CL, whereas no other covariates were retained in the final model. Although prior studies in GvHD have reported markedly lower CL compared with MF,^30^ our results did not show significant differences in ruxolitinib PK between underlying disease groups. The magnitude of the CYP inhibitors on ruxolitinib CL is consistent with previous PK and popPK studies showing that strong CYP3A4 or dual CYP2C9/CYP3A4 inhibition reduces ruxolitinib CL by approximately 15–44%.^22^, ^30^, ^40^ The clinical relevance of this interaction is illustrated by our simulations in patients treated with 10 mg ruxolitinib twice daily, in whom 39% of simulated individuals without and 85% of those receiving strong CYP3A or dual CYP3A/2C9 inhibitors exceeded the reported *C*_min_ toxicity threshold of 21 ng/mL.^30^ This threshold should be interpreted with caution, as it was derived from a relatively small GvHD cohort, was mainly associated with grade 1–2 adverse events, and did not include any grade 4–5 events. Nevertheless, these results suggest that standard dosing frequently yields concentrations associated with an increased risk of adverse events, particularly in patients treated with azole antifungals.

Importantly, our exploratory PK/PD analyses suggested that higher exposure was related to an increased risk of toxicity for *C*_min_ and AUC_24_ in the overall population. Whilst the limited event numbers preclude the identification of a definitive threshold, the findings suggest that AUC_24_ and *C*_min_ may represent useful predictive markers of exposure–toxicity in clinical practice. By contrast, the exposure–efficacy relationship was found to be inconclusive: no significant relationship between ruxolitinib exposure and severe disease activity was identified, either in the overall population or within individual disease subgroups. The subjective nature of the efficacy endpoints, together with the limited sample size, likely resulted in insufficient statistical power to detect a significant association. Overall, our results are broadly consistent with previous analyses for ruxolitinib with respect to exposure–toxicity patterns, while a robust exposure–efficacy relationship could not be demonstrated in this heterogeneous real‐world cohort.^20^, ^30^

The limitations of this study should be acknowledged. First, although the detailed PK sub‐study improved characterization of the structural model, most patients contributed only sparse samples obtained in routine care. Second, some clinically relevant covariates were incompletely captured: markers of inflammation, disease‐specific biomarkers, and additional surrogate markers of organ dysfunction could not be fully explored. In particular, given the heterogeneity of the cohort beyond MPN (e.g., GvHD), only a limited number of MPN‐SAF total symptom Score (MPN‐SAF TSS; MPN 10)^41^ were available, which did not allow their use in the analysis. In addition, population heterogeneity, while reflective of real‐world practice, limited indication‐specific inferences due to small subgroup sizes and potential disease‐related differences. Drug–drug interactions were driven predominantly by azole antifungals. Other drug classes may affect ruxolitinib exposure but were not sufficiently represented to be assessed. Third, the sample size remains limited, and the numbers of documented severe disease or toxicity were insufficient to precisely quantify exposure–response relationships and to define robust therapeutic intervals with established efficacy or toxicity thresholds. The PhGA score is a global measure that may be insufficiently sensitive to capture exposure‐related changes in disease activity. Late onset adverse events were probably under‐reported and may have attenuated exposure–toxicity relationships. Variations in treatment duration, dose adjustments, and adherence over time probably added further variability. These limitations probably contributed to imprecision in the PK/PD analyses. Eventually, neither external validation nor internal data‐split validation was performed and extrapolation of these findings to other settings and populations should be made with caution. Despite these limitations, our popPK model provided an adequate description of ruxolitinib concentrations across a wide range of dosing regimens and clinical situations. The simulated steady‐state profiles and exposure distributions were consistent with previous ruxolitinib studies and help future studies on TDM implementation in routine practice.

In conclusion, this prospective real‐world study provides one of the first detailed characterizations of ruxolitinib PK in a heterogeneous hematologic patient population. The model confirms the potent impact of strong CYP3A4 and dual CYP3A4/CYP2C9 inhibitors on CL and exposure and shows moderate‐to‐high between‐subject variability. The exploratory PK/PD results support an exposure–toxicity relationship, whereas no evident exposure–efficacy signal could be identified. These findings are particularly important given the pronounced increase in exposure among patients receiving CYP3A or dual CYP3A/2C9 inhibitors, in whom simulated *C*_min_ frequently exceeded a previously proposed toxicity threshold. This is highly relevant in clinical practice as patients with hematologic disorders are often co‐prescribed azole antifungals. Taken together, these data support a potential role for TDM to assist dose adjustment in patients, particularly those treated with strong CYP inhibitors or with other factors that may alter metabolism. Future studies in larger cohorts should define the therapeutic interval based on thresholds for efficacy and toxicity and incorporate active metabolites. These steps are needed to develop routine TDM strategies that improve the safety and effectiveness of ruxolitinib. A similar approach should be extended beyond ruxolitinib, as other clinical JAK2 inhibitors used in MPN (e.g., momelotinib)^42^ also show marked between‐subject variability, exposure–response relationships, with clinically relevant contributions from active metabolites.

## FUNDING

This study was funded by the Swiss National Science Foundation (Grant number: 10000892).

## CONFLICT OF INTEREST

SCM reports advisory board/consultancy activity with Incyte, Novartis, GSK, Orpha Swiss, Bristol Myers Squibb (BMS), Celgene, AbbVie, and Takeda; honoraria from Novartis, BMS, Celgene, Incyte, Orpha Swiss, AbbVie, GSK, and Takeda; research funding from Ajax Therapeutics Inc.; other financial disclosures from Novartis, Orpha Swiss, AbbVie, Amgen, and Janssen; and patents/licensing with the University of Basel/Novartis. All other authors declared no competing interests for this work.

## AUTHOR CONTRIBUTIONS

J.T. wrote the manuscript. J.T., M.G., L.A.D, and F.R.G. designed the research. J.T., S.C.M., F.G., E.H., M.N.‐H., J.P.H., J.R.P., D.H., C.B., L.A.D, M.G., and F.R.G performed the research. J.T. and M.G. analyzed the data.

## Supporting information


Data S1
