# Supplementary material for: Ruxolitinib Pharmacokinetics and Exposure–Toxicity Relationship in Hematologic Malignancies and Immune‐Mediated Diseases: A Prospective Observational Study
Source: Clin Pharmacol Ther. 2026 Jun 24;120(3):753–61. doi: 10.1002/cpt.70367 (PMC13339044; doi:10.1002/cpt.70367)
Supplement: Supplementary file 1 — Data S1 [file CPT-120-753-s001.docx]

**Supplementary material**

**Table S1. Summary of univariate covariate analysis**

| **PK parameter** | **Covariate** | **∆2LL**  (p-value: * < 0.05, ** < 0.01, *** < 0.001) | **β_cov_, RSE (%)** |
| --- | --- | --- | --- |
| CL | Age | -0.43 | 0.08 (207) |
|  | Sex | -1.03 | -0.1 (123) |
|  | Body weight | -0.88 | -0.03 (695) |
|  | Height | -0.7 | 0.05 (5421) |
|  | BMI | -1.11 | 0.06 (662) |
|  | CYP inhibitors (none vs moderate vs strong vs missing data)^a^ | -11.13 (***) |  |
|  | - Moderate CYP3A inhibitors^b^ |  | 0.97 (15) |
|  | - strong CYP3A^c^, dual CYP2C9 and CYP3A inhibitor^d^ |  | -0.24 (51) |
|  | - Missing data^e^ |  | 0.25 (154) |
|  | CYP inhibitors (strong vs none/missing data/moderate)^f^ |  |  |
|  | - Strong CYP3A, dual CYP2C9 and CYP3A inhibitor | -12.07 (***) | -0.46 (24) |
|  | Underlying disease^g^ | -1.4 |  |
|  | - GvHD |  | 0.063 (195) |
|  | - Other hematological diseases^h^ |  | -0.06 (319) |
|  | - Missing data |  | 0.29 (112) |
| V | Age | -0.59 | -0.08 (362) |
|  | Sex | -5.04 (*) | 0.24 (58) |
|  | Body weight (estimated exponent) | -14.2 (***) | 0.75 (30.7) |
|  | Body weight (fixed exponent) | -13.6 (***) | 1 (FIX) |
|  | Height | -6.63 (**) | 1.91 (64) |
|  | BMI | -10.8 (***) | 1.08 (28) |

BMI, body mass index; CL, clearance; CYP, Cytochrome P450; GvHD, graft-versus-host disease; V, volume of distribution; β_cov_, fixed effect of the covariate; ∆2LL: difference between two nested models objective function values.

^a^ No-CYP inhibitor is the reference group.

^b^ Includes isavuconazole, letermovir, and verapamil.

^c^ Includes posaconazole.

^d^ Includes fluconazole.

^e^ no data available on CYP inhibitor status.

^f^ None/missing/moderate is the reference group.

^g^ MPN (includes myelofibrosis, polycythemia vera, essential thrombocythemia) is the reference group.

^h^ Includes VEXAS syndrome, APECED syndrome, HLH

Regarding CYP inhibition in univariate testing, clearance in the rich analysis was similar in the moderate CYP3A inhibitors group (n = 8 samples) and the no inhibitor group (n = 181 samples), and was poorly estimated in the missing data category (n = 8 samples). Therefore, these categories were pooled together, and the reduced analysis evaluated strong CYP3A and dual CYP3A/2C9 inhibitors versus all other categories. During backward elimination, removing either the strong CYP inhibitor effect or the body-weight effect similarly worsened the fit.

**Table S2. Distribution of dosing regimens across samples and patients.**

| **Category** | **Dosing Regimen** | **Number of samples, n (%)**  **(n = 221)** | **Number of patients, n (%)**  **(n = 77)** |
| --- | --- | --- | --- |
| **Regular Symmetrical Dosing** |  |  |  |
|  | 5 mg QD | 6 (3) | 5 (7) |
|  | 5 mg BID | 20 (9) | 15 (20) |
|  | 10 mg BID | 69 (31) | 34 (44) |
|  | 15 mg BID | 16 (7) | 7 (9) |
|  | 20 mg QD | 1 (0.5) | 1 (1) |
|  | 20 mg BID | 67 (30) | 18 (23) |
|  | 25 mg BID | 17 (8) | 3 (4) |
| **Asymmetrical Dosing**  **(Morning - Midday - Evening)** |  |  |  |
|  | 10 mg - 0 mg - 5 mg | 1 (0.5) | 1 (1) |
|  | 10 mg - 0 mg - 15 mg | 9 (4) | 2 (3) |
|  | 10 mg - 0 mg - 20 mg | 1 (0.5) | 1 (1) |
|  | 15 mg - 0 mg - 20 mg | 1 (0.5) | 1 (1) |
|  | 20 mg - 0 mg - 10 mg | 1 (0.5) | 1 (1) |
| **Extended-Interval Dosing** |  |  |  |
|  | 5 mg every 72 hours | 2 (1) | 1 (1) |
|  | 10 mg every 48 hours | 7 (3) | 1 (1) |
|  | 15 mg three times a week | 3 (1) | 1 (1) |

BID, twice daily; QD, once daily.

**Table S3. Reported adverse events.**

| **Categories** | **Reported adverse events** | |
| --- | --- | --- |
|  | **Samples, n (%)**  **(n = 221)** | **Patients, n (%)**  **(n = 77)** |
| **No adverse events** | 120 (54) | 49 (64) |
| **Any adverse events** | 101 (46) | 28 (36) |
| Metabolism and nutrition disorders^a^ | 47 (21) | 8 (10) |
| Blood and lymphatic system disorders^b^ | 46 (21) | 15 (19) |
| Hepatobiliary disorders^c^ | 24 (11) | 4 (5) |
| Cardiovascular disorders^d^ | 21 (10) | 3 (4) |
| Neoplasms benign, malignant and unspecified^e^ | 21 (10) | 4 (5) |
| Infections and infestations^f^ | 19 (8) | 5 (6) |
| Gastrointestinal disorders^g^ | 16 (7) | 3 (4) |
| Musculoskeletal and connective tissue disorders^h^ | 8 (4) | 1 (1) |
| Respiratory, thoracic and mediastinal disorders^i^ | 7 (3) | 1 (1) |
| Nervous system disorders^j^ | 1 (<1) | 1 (1) |
| General disorders^k^ | 1 (<1) | 1 (1) |

^a^ Includes weight gain, weight loss, hypercholesterolemia, and dyslipidemia.

^b^ Includes anemia, cytopenia, leukopenia, neutropenia, thrombocytopenia.

^c^ Includes alanine aminotransferase increased, aspartate aminotransferase increased.

^d^ Includes thromboembolic event, myocardial infarction.

^e^ Includes non-melanoma skin cancer (NMSC).

^f^ Includes *herpes zoster* reactivation, esophageal candidiasis, meningitis.

^g^ Includes abdominal pain, bloating, diverticulitis.

^h^ Includes myalgia.

^i^ Includes dyspnea.

^j^ Includes tremor.

^k^ Includes asthenia.


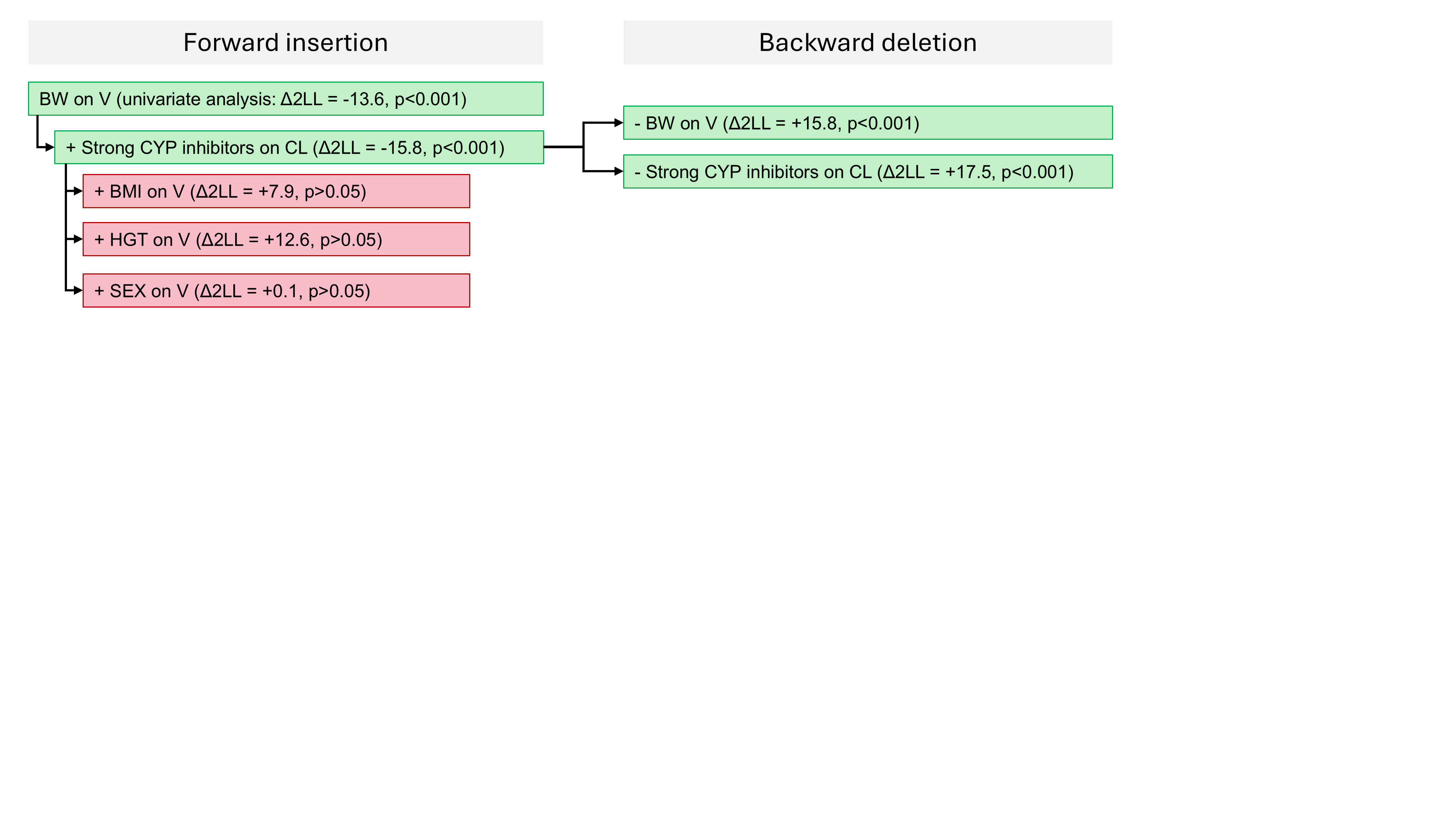


**Figure S1. Summary of forward insertion and backward deletion covariate analysis.** Strong CYP inhibitors include strong CYP3A and dual CYP2C9 and CYP3A inhibitors. Green boxes indicate covariates retained in the model, while red boxes those not retained in the model. BMI: body mass index; BW: body weight; CL: clearance; CYP: Cytochrome P450; HGT: Height; V: volume of distribution; ∆2LL: difference between two nested models objective function values.





**Figure S2. Goodness-of-fit plots**. Upper panel: scatter plots of individual weighted residuals (IWRES) versus time after dose (left) and versus individual predictions for the final model (right). The solid horizontal line denotes the zero-reference line. Lower panel: scatter plots of observed ruxolitinib plasma concentrations versus individual predictions on linear (left) and log–log (right) scales. The dashed black lines illustrate linear regression fit. The line of identity is printed in solid black line. Red dots represent censored data, and the dashed grey line indicates the lower limit of quantification (LLOQ: 0.5 ng/mL) of the analytical method.


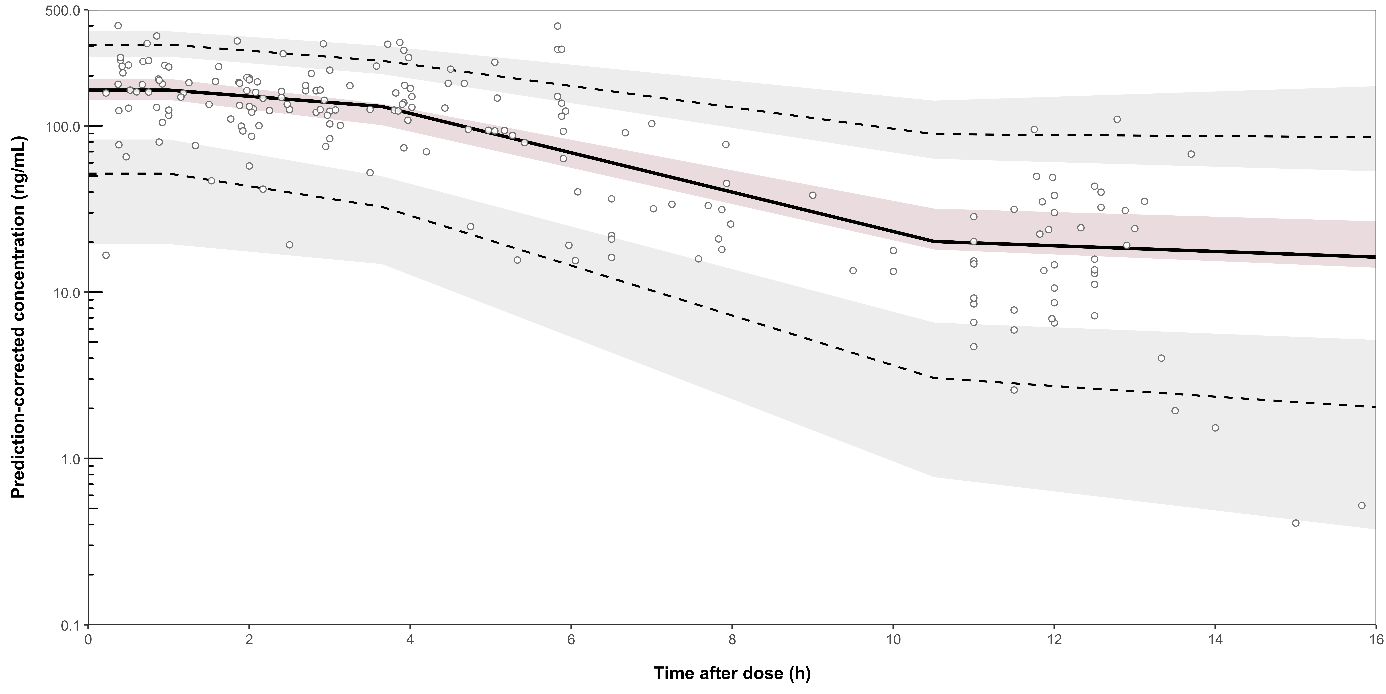


**Figure S3. Prediction-corrected visual predictive check (pcVPC) for the final ruxolitinib model, presented on a semi-logarithmic scale.** Open circles represent prediction-corrected observed plasma concentrations, with solid and dashed lines showing the corresponding median and 90% prediction intervals (PI_90%_), respectively. Red and grey shaded areas depict the model-predicted 90% confidence intervals around the simulated median and PI_90%_, respectively.


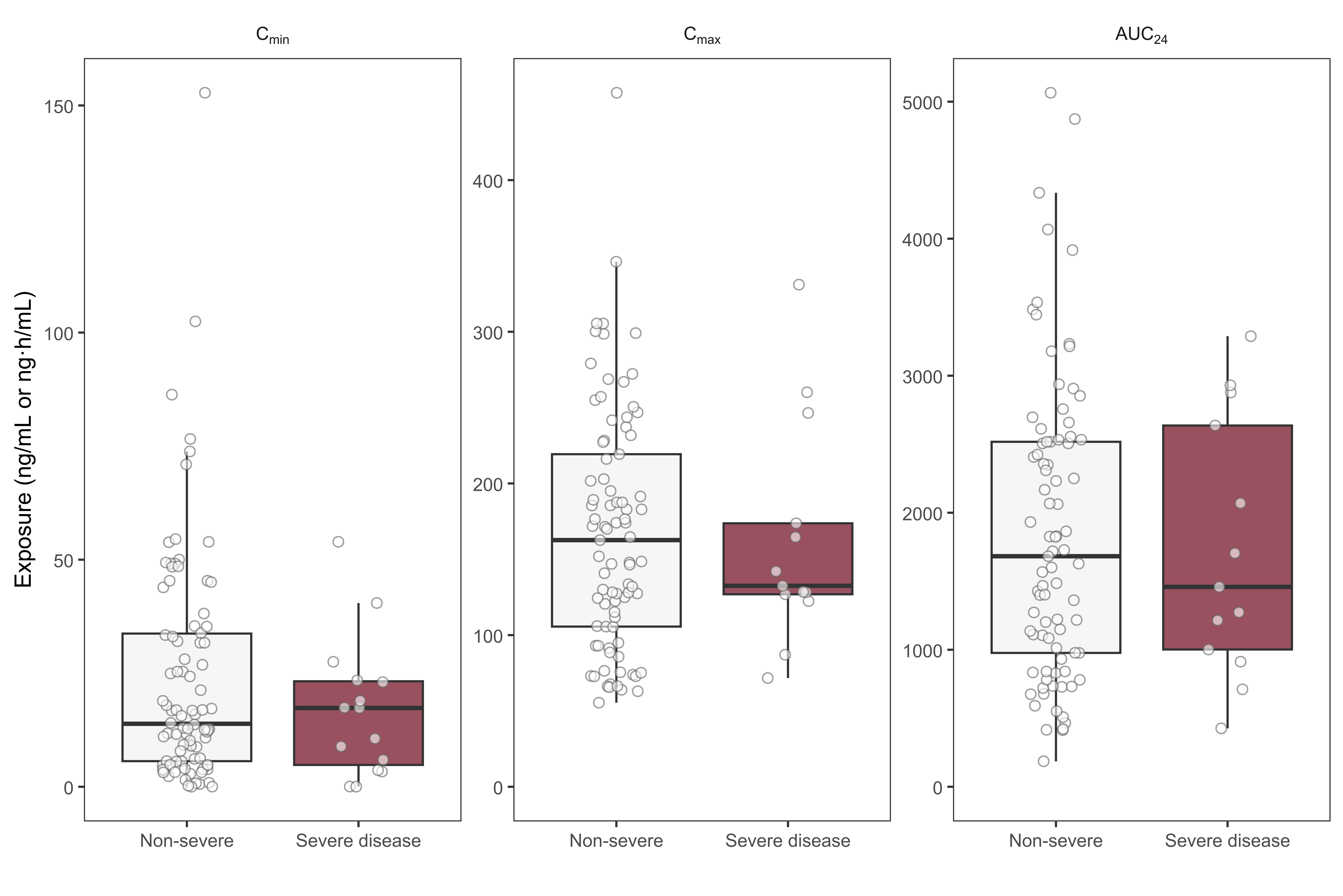


**Figure S4. Exposure–efficacy relationship for ruxolitinib** **in the overall population.** Boxplots show model-predicted steady-state C_min_, C_max_ and AUC_24_, according to efficacy status. Points represent individual observations. Boxes indicate median and interquartile range (IQR), and whiskers extend to 1.5× IQR. AUC_24_, area under the curve over the dosing interval of 24h; C_max_, peak plasma concentrations; C_min_, trough concentrations.
